# Synthesis of new Ln_4_(Al_2_O_6_F_2_)O_2_ (Ln = Sm, Eu, Gd) phases with a cuspidine-related structure

**DOI:** 10.1107/S205225251801744X

**Published:** 2019-01-01

**Authors:** Aroa Morán-Ruiz, Aritza Wain-Martin, Alodia Orera, María Luisa Sanjuán, Aitor Larrañaga, Peter R. Slater, Maribel Arriortua

**Affiliations:** a Universidad del País Vasco (UPV/EHU), Facultad de Ciencia y Tecnología, Barrio Sarriena S/N, Leioa, Vizcaya 48940, Spain; b Instituto de Ciencia de Materiales de Aragón (CSIC - Universidad de Zaragoza), C/ Pedro Cerbuna 12, Zaragoza 50009, Spain; c University of Birmingham, School of Chemistry, Birmingham B15 2TT, UK; d BCMaterials (Basque Centre for Materials, Applications and Nanostructures), Bld. Martina Casiano, 3rd. Floor, UPV/EHU Science Park, Barrio Sarriena S/N, Leioa, Vizcaya 48940, Spain

**Keywords:** cuspidine-type rare-earth aluminates, fluorination, X-ray diffraction, poly(vinyl­idene difluoride), Raman spectroscopy

## Abstract

The first fluorination of the cuspidine-related phases of Ln_4_(Al_2_O_7_□)O_2_ (where Ln = Sm, Eu, Gd) is reported. A low-temperature reaction with poly(vinyl­idene difluoride) lead to the fluorine being substituted in place of oxygen and inserted into the vacant position between the dialuminate groups. X-ray photoelectron spectroscopy shows the presence of the F 1*s* photoelectron together with an increase in Al 2*p* and rare-earth 4*d* binding energies supporting F incorporation. Energy-dispersive X-ray spectroscopy analyses are consistent with the formula Ln_4_(Al_2_O_6_F_2_)O_2_, confirming that substitution of one oxygen by two fluoride atoms has been achieved. Rietveld refinements show an expansion in the cell upon fluorination and confirm that the incorporation of fluoride in the Ln_4_(Al_2_O_7_□)O_2_ structure results in changes in Al coordination from four to five. Thus, the isolated tetrahedral dialuminate Al_2_O_7_ groups are converted to chains of distorted square-based pyramids. These structural results are also discussed based on Raman spectra.

## Abstract 2

The success and effects of fluorination on the starting Ln_4_(Al_2_O_7_□)O_2_ (Ln = Sm, Eu, Gd) cuspidine-related structure were investigated. The incorporation of fluoride in this structure resulted in the conversion of the isolated tetrahedral dialuminate Al_2_O_7_ groups to chains of distorted square-based pyramids.

## 1. Introduction   

Minerals belonging to the cuspidine group have the general stoichiometry *M*_4_(Si_2_O_7_)*X*_2_ (*M* = divalent cation; *X* = OH, F, O), with Ca_4_(Si_2_O_7_)(OH,F)_2_ being the archetype compound. The cuspidine structure can be described as built up of chains of edge-sharing *M*O_7_/*M*O_8_ polyhedra running parallel to the *a* axis (in the *P*2_1_/*c* space group); the tetrahedral disilicate groups (Si_2_O_7_) interconnect with these ribbons through the vertices. The structural formula of cuspidine is better described as Ca_4_(Si_2_O_7_□)(OH,F)_2_ to directly show the vacant position between the disilicate groups. The filling of that position may convert the isolated pyrogroups into infinite chains of distorted trigonal bipyramids (Martín-Sedeño *et al.*, 2004 ▸).

Other systems also adopt this structural type, including the Ln_4_(Al/Ga)_2_O_9_ (Ln = rare-earth) type phases, which have attracted attention because of their ionic conductivity and thermal stability (Ghosh, 2015 ▸; Zhou *et al.*, 2014 ▸; Martín-Sedeño *et al.*, 2006 ▸; Morán-Ruiz *et al.*, 2018 ▸). In more recent years, the preparation and characterization of inorganic oxyfluorides have attracted significant interest. Thus, low-temperature fluorination methods can alter chemistry of the precursor oxide in different ways by charge compensation effects (Clemens & Slater, 2013 ▸). In particular, polymer reagents such as poly(vinyl­idine fluoride) and poly(tetra­fluoro­ethyl­ene) have been proven to be successful low-temperature fluorinating reagents, following the early work by Slater (2002 ▸) which illustrates the use of PVDF to prepare Ca_2_CuO_2_F_2_ and Sr_2_TiO_3_F_2_. Since then, a wide range of perovskite and related phases have been successfully fluorinated using this polymer route (Clemens *et al.*, 2014 ▸; Hancock *et al.*, 2012 ▸; Berry *et al.*, 2008 ▸; Heap *et al.*, 2007 ▸), and the method has been shown to be equally applicable to the fluorination of thin films (Kawahara *et al.*, 2017 ▸; Katayama *et al.*, 2016 ▸; Moon *et al.*, 2015 ▸). This earlier research has mainly focused on the fluorination of transition-metal containing materials, and so here we investigate the potential use for the fluorination of oxide systems that do not contain transition metals. In particular, given the recent studies on oxide ion/proton conductivity in La_4_(Ga_2−*x*_Ti_*x*_O_7+*x*/2_)O_2_, which illustrate the ability of the cuspidine structure to accommodate extra anions (Martín-Sedeño *et al.*, 2005 ▸), this would appear to be an ideal structure to examine the possible incorporation of fluoride. We have therefore investigated the fluorination of Ln_4_(Al_2_O_7_□)O_2_ to give new Ln_4_Al_2_O_9−*x*_F_2*x*_ (Ln = Sm, Eu, Gd) (0 ≤ *x* ≤ 1) phases. Here we report the results of these first low-temperature fluorination reactions of a range of rare-earth aluminate cuspidine-related phases. The introduction of fluorine (2 F^−^ replacing O^2−^) was achieved through a reaction with poly(vinyl­idene fluoride) (PVDF) as the fluorinating agent. We investigate the success and effects of fluorination on the starting structure by X-ray diffraction (XRD), X-ray photoelectron spectroscopy (XPS), ^27^Al solid-state nuclear magnetic resonance (NMR), Raman spectroscopy, scanning electron microscopy (SEM) and energy dispersive X-ray spectroscopy (EDX). The thermal stability of these samples after fluorination was evaluated in air through thermogravimetric analysis (TGA).

## 2. Experimental   

### 2.1. Powder preparation   

Starting precursor oxides of Ln_4_(Al_2_O_7_□)O_2_ (Ln = Sm, Eu, Gd) were prepared by the glycine nitrate combustion route using the appropriate quantities of metals and combustible substance as previously reported by Morán-Ruiz *et al.* (2018 ▸). The introduction of fluorine (2 F^−^ replacing O^7−^) into the Ln_4_(Al_2_O_7_□)O_2_ structure was achieved through a low-temperature (400°C) reaction with PVDF (Slater, 2002 ▸) as the fluorinating agent. Thus, fluorination was achieved by mixing the rare-earth aluminate phase with PVDF in a 1:1 mol ratio (precursor oxide: CH_2_CF_2_ monomer unit) and heating (80°C h^−1^) the mixture at 400°C for 12 h in air.

Since poly(tetra­fluoro­ethyl­ene) (PTFE) has also been shown to be a very good fluorinating reagent, we investigated the possibility of fluorination of Eu_4_(Al_2_O_7_□)O_2_ with PTFE under the same conditions. This gave similar results to the reaction with PVDF, with an observed expansion in the unit cell consistent with F incorporation.

### 2.2. Characterization techniques   

X-ray powder diffraction patterns were recorded with a Philips X’Pert-Pro diffractometer using graphite-monochromated Cu Kα_1,2_ radiation (λ_1_ = 1.5406 Å; λ_2_ = 1.5443 Å). The compounds were scanned between 15 and 90° (2θ) in 0.026° steps, counting 380 s per step. In addition, a Bruker D8 Advance Vårio diffractometer, equipped with a primary monochromator and a solid SolX detector, with energy discrimination optimized for such radiation (Cu *K*α_1_, λ_1_ = 1.5406 Å), were also used to improve the quality of the XRD data for structure refinement. The overall measuring time was ∼120 h per pattern to have good statistics over the 2θ angular range of 5–100° with a 0.02° step size. The fitting of the measured and calculated pattern structure refinement was carried out using the program *FullProf* (Rodríguez-Carvajal, 2011 ▸). Moreover, *Atoms62* software (Shape Software, 2005 ▸) was also used to illustrate the structure.

X-ray photoelectron spectroscopy (XPS) measurements were performed using an XPS spectrometer (SPECS). All XPS spectra were acquired using a monochromatic X-ray source producing Al *K*α radiation (*h*ν = 1486.6 eV) and recorded using a Specs Phoibos 150 analyser. An initial analysis of the elements present in the sample was carried out (wide scan: step energy 1 eV, dwell time 0.1 s, pass energy 80 eV) and individual high-resolution spectra were obtained (detail scan: step energy 0.1 eV, dwell time 0.1 s, pass energy 30 eV) with an electron take-off angle of 90°. The binding energies (BEs) were calibrated using the C 1*s* peak (BE = 284.6 eV) as an internal standard. The spectra were fitted by *CasaXPS* 2.3.16 software, modelling the properly weighted sum of Gaussian and Lorentzian component curves, after background subtraction according to Shirley.

The ^27^Al solid-state NMR spectra were recorded on a Bruker Avance III, at 9.4 T under magic angle (MAS) at 14 kHz using a Bruker probe head 4 mm MAS DVT X/Y/H. The ^27^Al MAS NMR were recorded at 104.27 MHz using a single-pulse sequence with a 4 µs rf pulse (π/2); the relaxation delay was 0.5 s and a total of 20 000 scans were accumulated. The ^27^Al chemical shifts were calibrated indirectly with Al(NO_3_)_3_.

For Raman scattering measurements a DILOR XY spectrometer with a CCD detector and 2 cm^−1^ of spectral resolution was used. The 514.5 nm line of an Ar^+^-ion laser was used as the excitation source, and the power output was kept below 20 mW after verifying that no changes were induced in the samples. A 50× microscope objective lens was used both for excitation and dispersed light collection. Some spectra were also collected in a WITEC Alpha 300M+ spectrometer working with 633 nm excitation. For each material, at least 3–4 representative spectra of different sample zones were recorded.

Thermogravimetric analyses were performed for all compositions on a TA Instruments SDT 2960 simultaneous DSC–TGA balance. The temperature was varied from room temperature up to 900°C at a heating rate of 3°C min^−1^ in air.

Compositional analysis was performed using an analytical scanning electron microscope (SEM, JEOL JSM-7000 F) with an electron microanalysis probe EDX (Oxford Pentafet energy dispersive X-ray analyzer). Samples were coated with a coal graphite layer (10 nm) deposited by evaporation (Quorum Q150T Sputter Coater) to provide electrical conductivity. Back-scattered electrons were measured at a 20 kV accelerating voltage and 5 × 10^−9^ A current. A measurement time of 100 s per point was established for data acquisition. EDX system calibration was performed by measuring the beam current on Ln_4_(Al_2_O_7_□)O_2_ and AlF_3_ as standards, to allow quantitative elemental analyses. The data processing was performed using Oxford *Inca* software. The characteristic emission lines used for the analysis were *K*α for Al and F, and *M*α for Sm, Eu and Gd. The morphologies of the powders were observed using secondary electrons at an accelerating voltage of 20 kV, a current of 1.1 × 10^−11^ A and a working distance of 9 mm. These samples were metallized by gold sputtering for better image definition.

## 3. Results and discussion   

**Figure 1:**
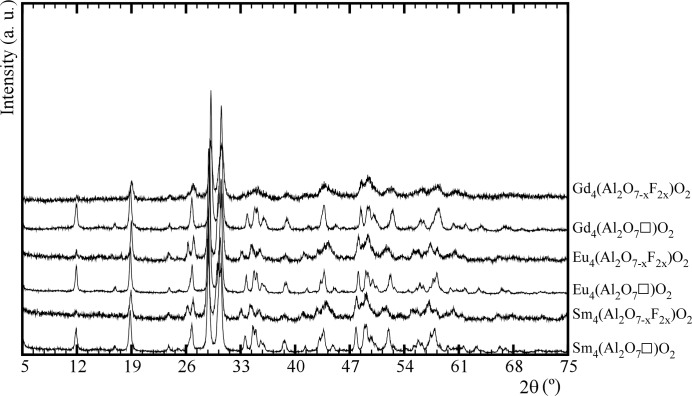
X-ray powder diffraction patterns recorded from materials of composition Ln_4_(Al_2_O_7_□)O_2_ (Ln = Sm, Eu, Gd) and their fluorinated derivatives.

The X-ray powder diffraction patterns recorded from Ln_4_(Al_2_O_7_□)O_2_ (Ln = Sm, Eu, Gd) and their new fluorinated derivatives are shown in Fig. 1 ▸. The XRD patterns show that all the samples consist of a single phase without impurities. Moreover, the fluorination induces a shift in peak position to lower angles corresponding to an increase in unit-cell sizes as the total anion content increases.

**Figure 2:**
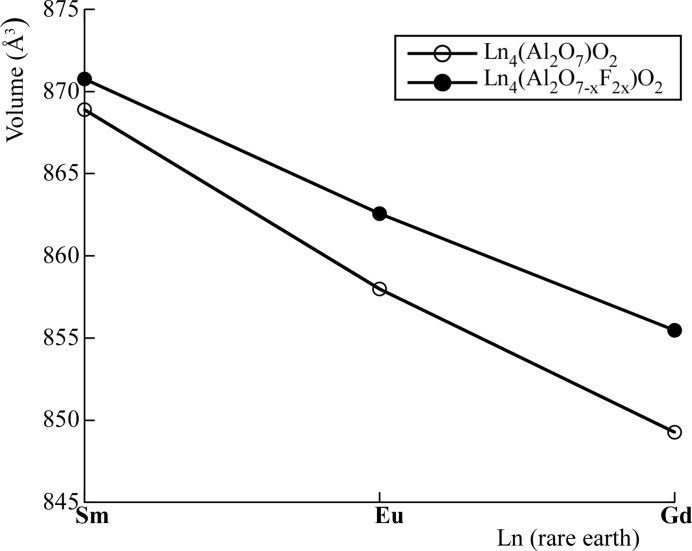
The volume changes between the pure oxides and their fluorinated derivatives.

The volumes recorded from the pure oxides and their fluorinated derivatives are graphically represented in Fig. 2 ▸. From the data in the graphic it can be seen that the fluorination leads to a significant increase in unit-cell parameters. The volume difference between the starting oxide and fluorinated oxides becomes more noticeable as the rare earth size decreases. Moreover, the cell parameters change in good agreement with the variation of the ionic radii of the rare-earth cations, with the largest cell volume observed for the Sm system and the smallest for the Gd system (Morán-Ruiz *et al.*, 2018 ▸) [Gd^3+^ (coordination number VII): 1.00 Å; Gd^3+^ (VIII): 1.05 Å; Eu^3+^ (VII): 1.01 Å; Eu^3+^ (VIII): 1.07 Å; Sm^3+^ (VII): 1.02 Å; Sm^3+^ (VIII): 1.08 Å].

**Figure 3:**
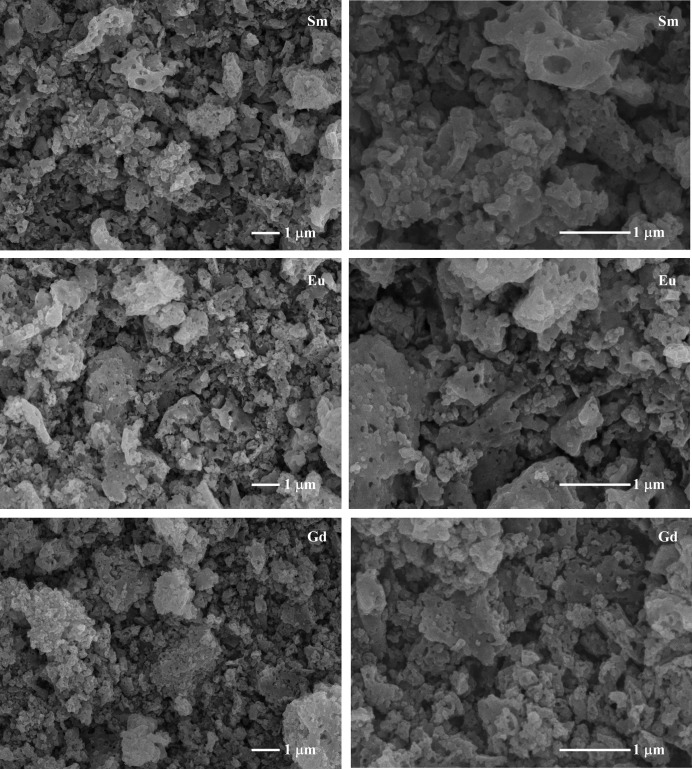
Micrographs of Sm_4_(Al_2_O_6_F_2_)O_2_, Eu_4_(Al_2_O_6_F_2_)O_2_ and Gd_4_(Al_2_O_6_F_2_)O_2_ phases prepared using a low-temperature fluorination route.

Representative SEM micrographs of the powder samples (as prepared and after fluorination at 400°C) are shown in Fig. 3 ▸. As observed, no significant differences can be seen in the morphology or the average particle size of the different samples in these images. All samples are composed of agglomerated sub-micrometre particles.

The chemical compositions of the obtained fluorinated oxides were analysed using SEM–EDX. The measured values of the elements were checked on different points to obtain the average composition. The atomic percentage concentrations of detected elements are listed in Table 1 ▸. For comparison, data were also collected for Eu_4_(Al_2_O_7_□)O_2_ fluorinated with half the molar equivalents of PVDF, in order to illustrate that F content can be controlled by the amount of polymer added.

These results indicate that the substitution of two fluorine atoms for one oxygen is satisfactorily achieved to obtain new Ln_4_Al_2_O_8_F_2_ (Ln = Sm, Eu, Gd) compositions. Examination of the fluorination with higher levels of PVDF led to no further increase in cell volume, illustrating the maximum F content had been reached. From these results it can be concluded that these cuspidine phases permit a maximum of two fluorine atoms per formula.

**Figure 4:**
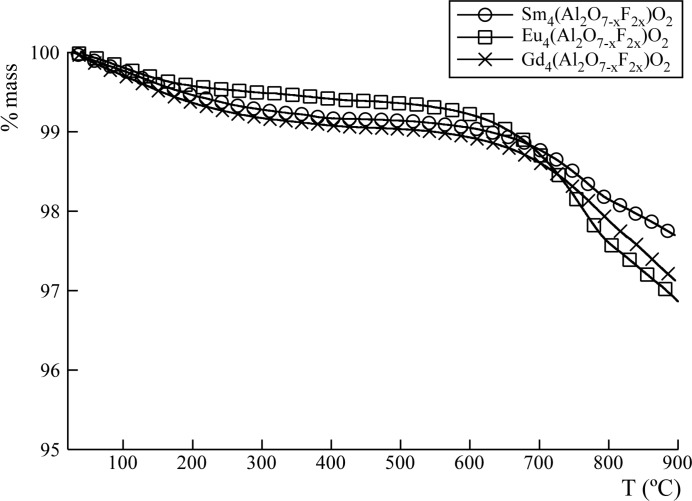
Thermogravimetric analysis of new Ln_4_(Al_2_O_6_F_2_)O_2_ (Ln = Sm, Eu, Gd) phases.

The samples were heated in a thermogravimetric analyzer in air at 900°C. The thermograms of all oxyfluorides are shown in Fig. 4 ▸. A decrease in mass with increasing temperature occurs between 550 and 900°C, which is associated with the loss of fluorine content due to the reaction with moisture in the air, leading to loss of HF and replacement by oxygen to reform the simple oxide system.

For all compositions, a gravimetric mass loss of ∼3% is observed. From these results the (O/F)_*z*_ relation is calculated (Table 2 ▸).

From the TGA data it can be concluded that the mass loss is not complete due to the low kinetic decomposition of these compounds. Preliminary studies show that the stabilization of the mass requires a long heating time (∼6 h) at 1000°C (see supporting information, Fig. S1). In order to obtain the total fluorine content remaining in each sample after treatment at 900°C, the residues were analyzed by EDX. The atomic percentage concentrations of detected elements are summarized in Table 3 ▸. The obtained data coincide with the calculated fluorine content loss.

**Figure 5:**
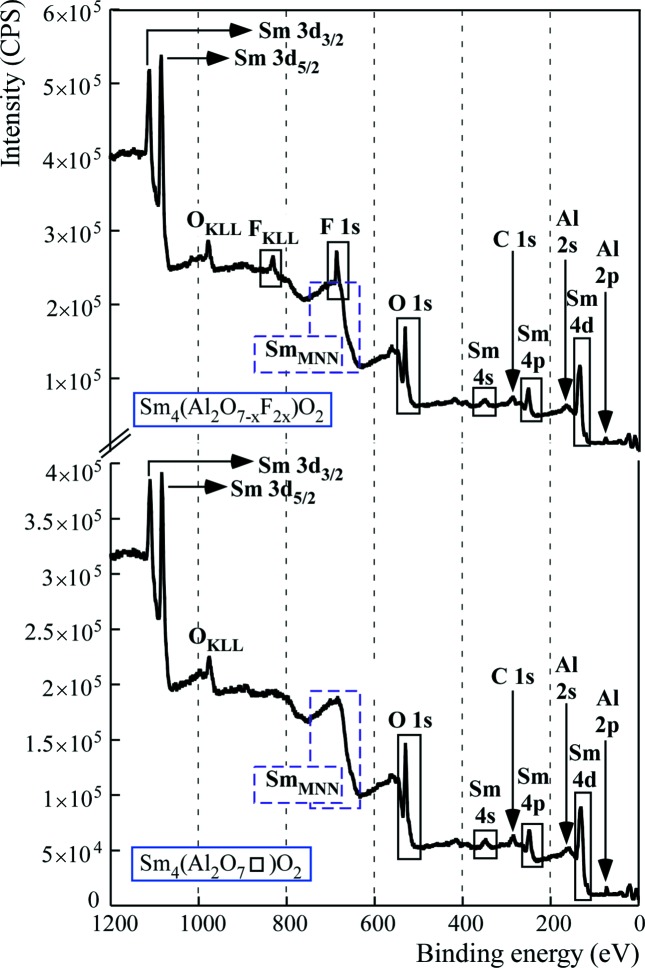
As an example, XPS survey spectra of the surface composition Sm_4_(Al_2_O_7_□)O_2_ and their new fluorinated derivative.

The success of the fluorination of rare-earth aluminates is also confirmed by XPS. A clear peak is observed in the analysed areas of the fluorinated oxides using a wide scan up to 1380 eV, attributable to an F 1*s* photoelectron (Fig. 5 ▸).

The resultant peak BEs before and after fluorination are presented in Table 4 ▸. In particular, we observe that fluorine incorporation induces an increase of the BEs of Al 2*p* and Ln 4*d* due to fluorine having a higher electronegativity than oxide.

**Figure 6:**
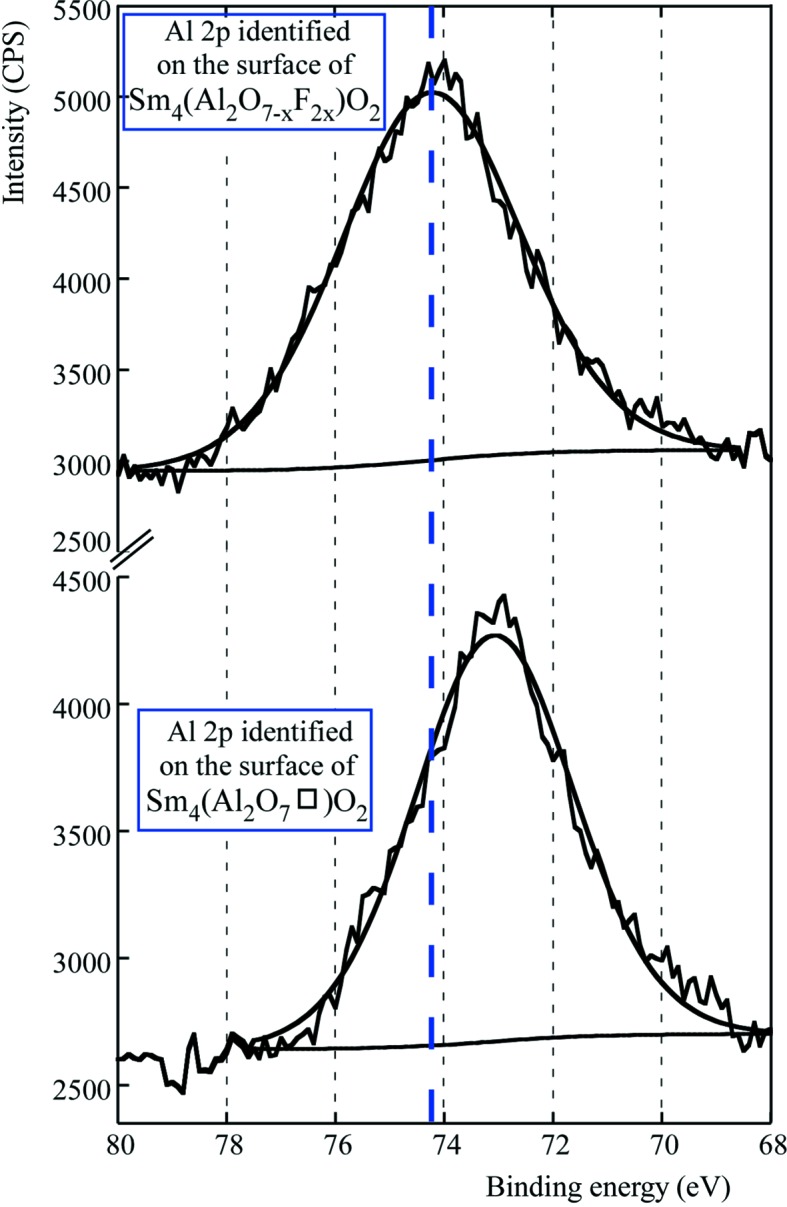
As an example, Al 2*p* spectral regions of the surface of Sm_4_(Al_2_O_7_□)O_2_ and their new fluorinated derivative showing a shift to higher binding energy upon fluorination.

This indicates greater electron transfer to fluorine, causing a decrease in the electron density at the cation and resulting in higher binding energy of the electrons from the core level of the cation (Dae-Min *et al.*, 2011 ▸). These peak-position shifts are observed in the high-resolution spectra of the Al 2*p* and Ln 4*d* spectral regions (Fig. 6 ▸).

The ^27^Al NMR spectra of the fluorinated samples and Ln_4_(Al_2_O_7_)O_2_ (Ln = Sm, Eu, Gd) are provided as supporting information (Fig. S2). It seems that the shape of the Ln_4_(Al_2_O_6_F_2_)O_2_ spectra changes compared with the Ln_4_(Al_2_O_7_)O_2_ spectra, which could be due to a modification of the coordination environment of Al^3+^ in the fluorinated derivatives. However, the obtained ^27^Al NMR data are not conclusive due to the paramagnetism of Sm, Eu and Gd rare-earth metals.

**Figure 7:**
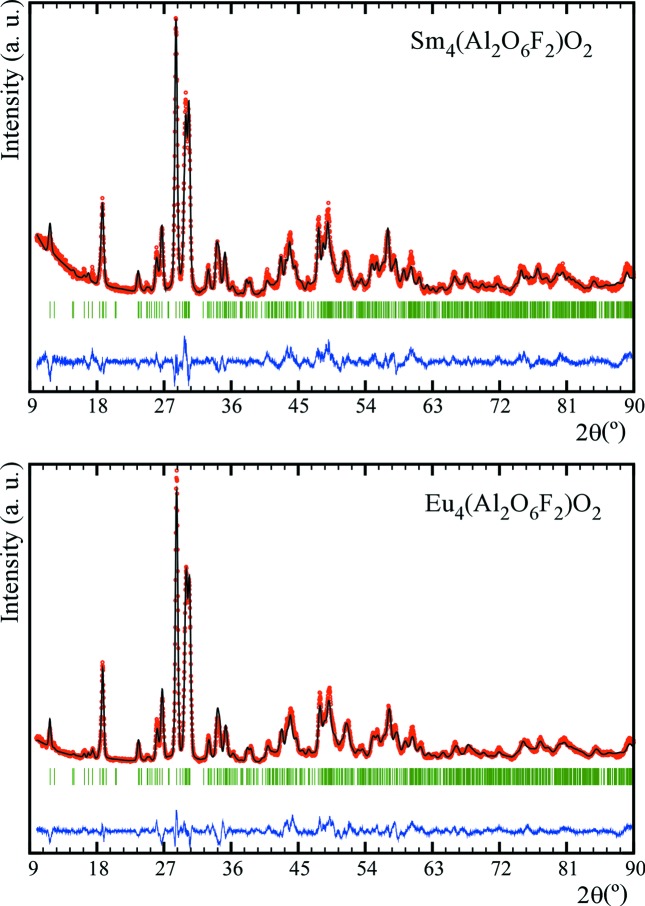
Rietveld refinement for new Sm_4_(Al_2_O_6_F_2_)O_2_ and Eu_4_(Al_2_O_6_F_2_)O_2_ cuspidine-related materials.

Full structural refinements of XRD data for Sm_4_Al_2_O_9−*x*_F_2*x*_ and Eu_4_Al_2_O_9−*x*_F_2*x*_ were carried out in the space group *P*2_1_/*c* by using the Sm_4_(Al_2_O_7_)O_2_ and Eu_4_(Al_2_O_7_)O_2_ structures as starting models, respectively. Refined cell and positional parameters, obtained bond distances and angles, and the bond valences are summarized in Tables S1–S11. The Rietveld fittings of the X-ray data are displayed in Fig. 7 ▸.

After the convergence of the overall parameters, the occupation of the bridge oxygen site O(5) was replaced by F(1) (Kendrick *et al.*, 2008 ▸) and an extra fluorine position, F(2) (Martín-Sedeño *et al.*, 2006 ▸), was added in the vacant anion site between two Al_2_O_7_ units, in order to account for the increase in anion content, and then refined. In both refinements, bond-length constraints were applied. The quality factors of the refinements are given in Table 5 ▸. It should be noted that distinguishing O and F by either X-ray or neutron diffraction is very difficult because of the nearly identical scattering factors. Therefore, the respective positions are commonly inferred by bond valence sum (BVS) calculations from the determined bond distances. In this respect, neutron diffraction data would lead to more accurate O/F positions and hence bond distances. However, Gd, Sm and Eu all show very strong neutron absorption, which makes such studies impractical. Therefore we have used BVS calculations based on the structures determined from the X-ray diffraction data. These calculations are in agreement with the assignment of the F positions proposed, which is further supported by the Raman results (see later). In addition, the BVS values that are calculated for the F(1) and F(2) sites, assuming O is present, show a critical deficit of valence charge in the oxygen atoms. These results add further weight to the conclusion that F occupies these sites.

The introduction of fluorine leads to the conversion of isolated *M*_2_O_7_□ groups into infinite chains of distorted square-based pyramids along the *a* axis, as observed for La_4_(Ti_2_O_8_)O_2_.

It is interesting to compare the present results with those of Si cuspidines of the *M*_4_(Si_2_O_7_)F_2_ type (Achary *et al.*, 2017 ▸), where the Si_2_O_7_ units are preserved and fluorine occupies the O(8) and O(9) sites instead of filling the anionic vacancies and substituting for bridge oxygen ions along the AlO_4_ chains. The different behaviour can be attributed to the larger size of Al cations compared with Si, and its higher ability to accommodate coordination numbers greater than four.

**Figure 8:**
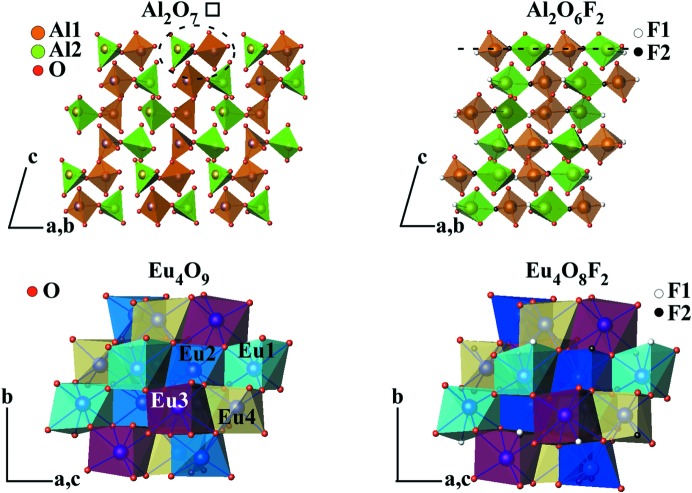
As an example, a polyhedral view of the Eu_4_(Al_2_O_7_□)O_2_ and Eu_4_(Al_2_O_6_F_2_)O_2_ phases obtained from the Rietveld refinement structural data using *Atoms62* software.

In summary, the structures of Ln_4_(Al_2_O_6_F_2_)O_2_ (Ln = Sm, Eu) are monoclinic (*P*2_1_/*c*) with two sites for fluorine between the aluminate groups. Thus, as observed from Figs. 8 ▸ and 9 ▸, the aluminium coordination changes from four to five. Because of low crystallinity, the Gd_4_(Al_2_O_7−x_F_2_)O_2_ diffractogram produces a poor signal, which limits its Rietveld refinement. This lower crystallinity is probably related to the fact that Gd is the smallest rare-earth metal and also the large volume change upon fluorination, which may have reduced the particle size/crystallinity. Considering Ln_4_(Al_2_O_6_F_2_)O_2_ (Ln = Sm, Eu) as representative structures of the obtained Ln_4_(Al_2_O_6_F_2_)O_2_ (Ln = Sm, Gd) compositions, similar results could be expected for the gadolinium sample.

**Figure 10:**
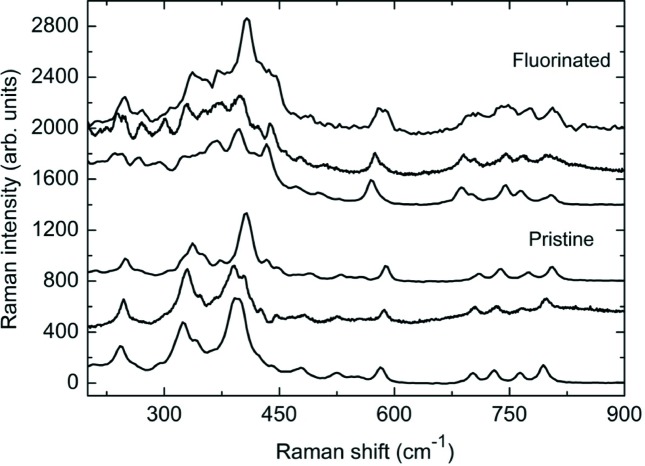
Raman spectra of the pristine Ln_4_(Al_2_O_7_□)O_2_ and Ln_4_(Al_2_O_6_F_2_)O_2_ samples. Bottom to top: Ln = Sm, Eu, Gd.

These structural assumptions have been further discussed based on Raman results. Raman spectra are shown in Fig. 10 ▸ for samples Ln_4_(Al_2_O_7_□)O_2_ (bottom set) and Ln_4_(Al_2_O_7−*x*_F_2*x*_)O_2_ (top set) (Ln = Sm, Eu, Gd). The relatively low intensity of all the spectra could be a priori attributed to the method of synthesis, where a low preparation temperature was used and thus low crystallinity was expected. The Raman spectra of the starting Ln_4_(Al_2_O_7_□)O_2_ materials are quite similar, since they are structurally akin, and are in good agreement with the bibliography (Hasdinor-Bin-Hassan, 2010 ▸). An evaluation of the whole spectra is beyond the scope of this work due to the complexity of the structure, so only the high-frequency region will be treated in detail. The as-prepared samples show four well defined bands between 700 and 800 cm^−1^ that can be unambiguously ascribed to Al—O stretching modes, since these have shorter bond distances than Ln—O. In the cuspidine structure, the existence of pyroaluminate units of [Al_2_O_7_] type suggests that it is appropriate to separate the expected modes into internal pyrogroup modes and lattice modes, although the covalent degree of the Al—O bond is lower than that of Si—O or P—O bonds in [Si_2_O_7_] or [P_2_O_7_] groups. Moreover, since these units are disconnected within the structure, correlation effects can be dismissed.

Following this approach, and taking into consideration the crystallographic results, the [Al_2_O_7_] units can be considered as consisting of two AlO_3_ pyramids connected by a bridging oxygen O′ [O(5) in Tables S2 and S3] in the form O_3_—Al—O′—Al—O_3._ Within this model, the expected modes can be divided into vibrational modes of the AlO_3_ pyramids and those of the Al—O′—Al bridge.

A regular pyramid with *C*3*v* symmetry would give two stretching modes in the region of study, one *A*_1_ mode and one *E* mode, consisting mainly of the vibration of the three oxygens of the pyramid along the Al−O bonds. However, since Al is located in a 4*e* site with very low local symmetry (*C*1), the pyramids must be considered as irregular, giving three *A* modes. On the other hand, the Al—O′—Al bridge is expected to give two stretching modes: one symmetric mode coming mainly from the vibration of Al atoms and one antisymmetric mode involving Al and O vibrations. The energy of the former will obviously depend on the cation and is found between 520–560 cm^−1^ in the case of [Ge_2_O_7_] (Saez-Puche *et al.*, 1992 ▸; Hanuza *et al.*, 2011 ▸) and [Ga_2_O_7_] (Kaminskii *et al.*, 2014 ▸) and 620–700 cm^−1^ for [Si_2_O_7_] (Achary *et al.*, 2017 ▸; Lecleach & Gillet, 1990 ▸), [P_2_O_7_] and [S_2_O_7_] (Kazuo, 2009 ▸). In our case, the Al vibration was expected to be around 600 cm^−1^ and could be tentatively ascribed to the intense band at 590 cm^−1^. Therefore, the only mode from the bridge in the high frequency region would be the antisymmetric mode. Although some authors have considered in analogous systems that the O′ is located in an inversion centre, thus yielding a Raman forbidden or very weak antisymmetric mode (Saez-Puche *et al.*, 1992 ▸), the approximation needs the angle of *X*—O′—*X* to be close to 180° and both *X*—O′ distances to be alike. These assumptions seem to be far from our case, where the *X*—O′—*X* angle is around 140°.

Since only four modes are observed in the high-frequency region of the Ln_4_(Al_2_O_7_□)O_2_ sample, the model that best fits our data is that of two irregular but similar pyramids, which would give three stretching modes, connected by an Al—O′—Al bridge whose antisymmetric mode would supply the required fourth mode.

The model of the isolated [Al_2_O_7_] units is not valid anymore for the fluorinated samples, where F is proposed to be located in the interstitial positions between these units as well as substituting for the O′ in the bridge. Thus, AlO_3_F_2_ quasi-square pyramids sharing F vertices form infinite chains along the *a* axis (see Fig. 10 ▸). By applying the point group *C*2*v* symmetry operations to the constituent atoms of the pyramid (two F and two O atoms in the base and one apical O_ap_), five stretching modes are expected in the high frequency region, considering that all the pyramids are equivalent: three A_1_ (Al + O_ap_, F, O), one B_1_ (Al + F) and one B_2_ (Al + O). This number of modes is in good agreement with what we observe in the spectra of the fluorinated samples, where five modes are found in the 650–820 cm^−1^ region. The agreement with the experimental observation suggests that correlation effects, if present, result in almost degenerate modes that remain unresolved because of the spectral broadening. Regarding the symmetrical mode of the Al—O′—Al bridge in the pristine samples, its position shifts from 590 to 570 cm^−1^ upon fluorination, which would agree with the substitution of the O′ bridge by F, supporting the assumption from the structural studies that F is located in this site.

Therefore, the Raman measurements are consistent with the crystallographic model proposed for fluorinated Ln_4_(Al_2_O_6_F_2_)O_2_ cuspidines.

## 4. Conclusions   

In summary, new Ln_4_(Al_2_O_6_F_2_)O_2_ (Ln = Sm, Eu, Gd) phases with a cuspidine-related structure have been synthesized using a low-temperature fluorination route, a technique that uses Ln_4_(Al_2_O_7_)O_2_ as the oxide precursor and poly(vinyl­idene difluoride) as the fluorination agent. The results illustrate the versatility of this fluorination route for the synthesis of new oxide–fluoride systems. The Raman measurements are consistent with the crystallographic model proposed for new fluorinated Ln_4_(Al_2_O_6_F_2_)O_2_ cuspidines: the incorporation of fluorine in the Ln_4_(Al_2_O_7_□)O_2_ structure results in Al coordination changes from four to five, which allows the conversion of isolated Al_2_O_7_□ groups into infinite chains of distorted square-based pyramids.

## Supplementary Material

Crystal structure: contains datablock(s) global, eu4al2o8f2, sm4al2o8f2. DOI: 10.1107/S205225251801744X/yc5017sup1.cif

Structure factors: contains datablock(s) eu4al2o8f2. DOI: 10.1107/S205225251801744X/yc5017eu4al2o8f2sup2.hkl

Structure factors: contains datablock(s) sm4al2o8f2. DOI: 10.1107/S205225251801744X/yc5017sm4al2o8f2sup3.hkl

Supporting tables and figures. DOI: 10.1107/S205225251801744X/yc5017sup4.pdf

CCDC references: 1886577, 1886578

## Figures and Tables

**Figure 9:**
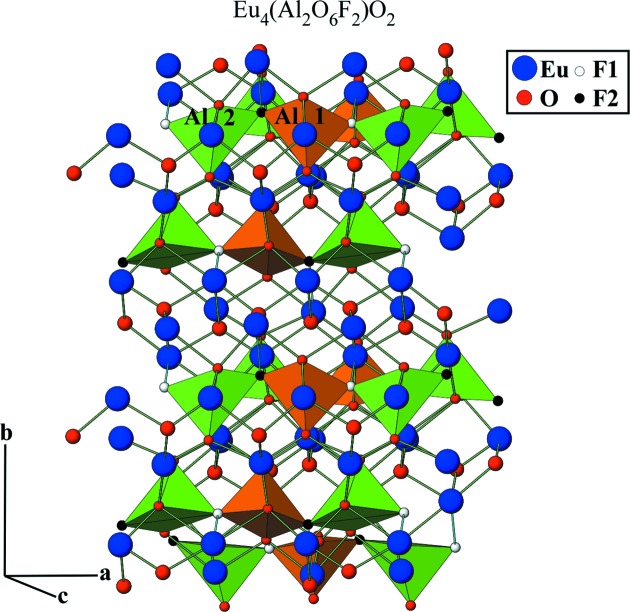
As an example, a simplified representation of the new Eu_4_(Al_2_O_6_F_2_)O_2_ phase structure obtained from the Rietveld refinement structural data using *Atoms62* software.

**Table 1:** Chemical compositions (at.%) of fluorinated oxides obtained under various synthetic conditions The use of plasma-cleaning could reduce the fluorine content near to 12.5 at.%.

Sample	Ln†	Al	F
Sm_4_(Al_2_O_7−*x*_F_2*x*_)O_2_	24.0 (2)	12.2 (2)	13.4 (3)
Eu_4_(Al_2_O_7−*x*_F_2*x*_)O_2_	25.0 (4)	12.2 (3)	13.1 (1)
Gd_4_(Al_2_O_7−*x*_F_2*x*_)O_2_	25.6 (2)	12.6 (3)	13.6 (2)
Eu_4_(Al_2_O_7−*x*_F_2*x*_)O_2_ ‡	23.2 (1)	12.0 (2)	6.9 (1)
Eu_4_(Al_2_O_7−*x*_F_2*x*_)O_2_ §	22.7 (2)	11.5 (1)	12.7 (2)
Eu_4_(Al_2_O_7−*x*_F_2*x*_)O_2_ ¶	23.0 (1)	11.5 (1)	12.3 (1)
Theoretical Ln_4_(Al_2_O_6_F_2_)O_2_	25	12.5	12.5

† Ln = Sm, Eu, Gd.

‡ Fluorination reaction using ½ PVDF [equivalent to 1 F (*x* = 0.5) incorporation].

§ Fluorination reaction using poly(tetra­fluoro­ethyl­ene) (PTFE).

¶ Fluorination reaction using ½ PTFE.

**Table 2:** Fluorine content loss calculated from the gravimetric mass loss (550–900°C)

Sample	*x*
Sm_4_(Al_2_O_7−*x*_F_2*x*_)O_2_	0.52
Eu_4_(Al_2_O_7−*x*_F_2*x*_)O_2_	0.93
Gd_4_(Al_2_O_7−*x*_F_2*x*_)O_2_	0.72

**Table 3:** Chemical compositions (at.%) of the TGA residues of fluorinated oxides

Residue	Ln† (at.%)	Al (at.%)	F (at.%)
Sm_4_(Al_2_O_7−*x*_F_2*x*_)O_2_	24.8 (3)	12.6 (3)	6.6 (3)
Eu_4_(Al_2_O_7−*x*_F_2*x*_)O_2_	26.9 (1)	12.7 (1)	1.0 (1)
Gd_4_(Al_2_O_7−*x*_F_2*x*_)O_2_	27.4 (3)	11.5 (3)	2.8 (6)

† Ln = Sm, Eu, Gd.

**Table 4:** XPS analysis results of detected elements for the surface of the obtained Ln_4_(Al_2_O_7−*x*_F_2*x*_)O_2_ compositions

Sample	Ln 4*d* † (BE, eV)	Al 2*p* (BE, eV)	F 1*s* (BE, eV)
Sm_4_(Al_2_O_7_)O_2_	131.7	73.1	–
Sm_4_(Al_2_O_7−*x*_F_2*x*_)O_2_	133.1	74.3	685.0
Eu_4_(Al_2_O_7_)O_2_	135.5	73.1	–
Eu_4_(Al_2_O_7−*x*_F_2*x*_)O_2_	137.0	74.3	686.5
Gd_4_(Al_2_O_7_)O_2_	141.9	73.1	–
Gd_4_(Al_2_O_7−*x*_F_2*x*_)O_2_	142.8	74.3	685.3

† Ln = Sm, Eu, Gd.

**Table 5:** The quality of refinements performed on new fluorinated oxides

Samples	Sm_4_Al_2_O_9−*x*_F_2*x*_	Eu_4_Al_2_O_9−*x*_F_2*x*_
χ^2^	3.7	5.7
*R*_Bragg_	12.8	11.7
*R*_f_	10.6	9.2
*R*_p_	10.9	10.2
*R*_wp_	13.6	12.8
*R*_exp_	7.06	5.39
