# Supplementary material for: Synthesis of new Ln4(Al2O6F2)O2 (Ln = Sm, Eu, Gd) phases with a cuspidine-related structure
Source: IUCrJ. 2019 Jan 1;6(Pt 1):128–35. doi: 10.1107/S205225251801744X (PMC6327175; doi:10.1107/S205225251801744X)
Supplement: Supplementary file 4 [file m-06-00128-sup4.pdf]

IUCrJ

Volume 6 (2019)

Supporting information for article:

Synthesis of new Ln4(Al2O6F2)O2 (Ln = Sm, Eu, Gd) phases with a cuspidine-related structure

Aroa Morán-Ruiz, Aritza Wain-Martín, Alodia Orera, María Luisa Sanjuán, Aitor Larrañaga, Peter R. Slater and Maribel Arriortua

Supporting information

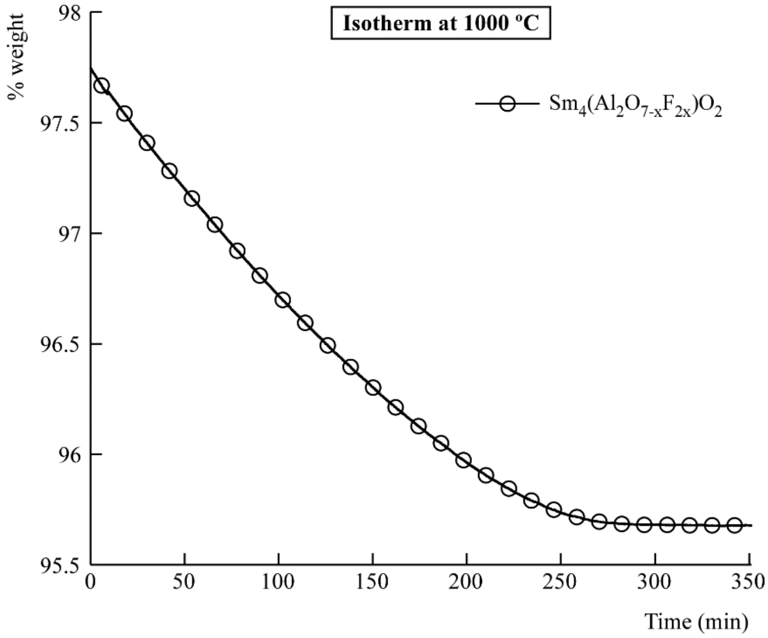

Figure S1. Thermogravimetric analysis (TGA) isotherm of $Sm_4(Al_2O_6F_2)O_2$ phase at 1000 ºC. The isotherm starting point corresponds to the weight loss observed at 1000 ºC (time= 0 min).

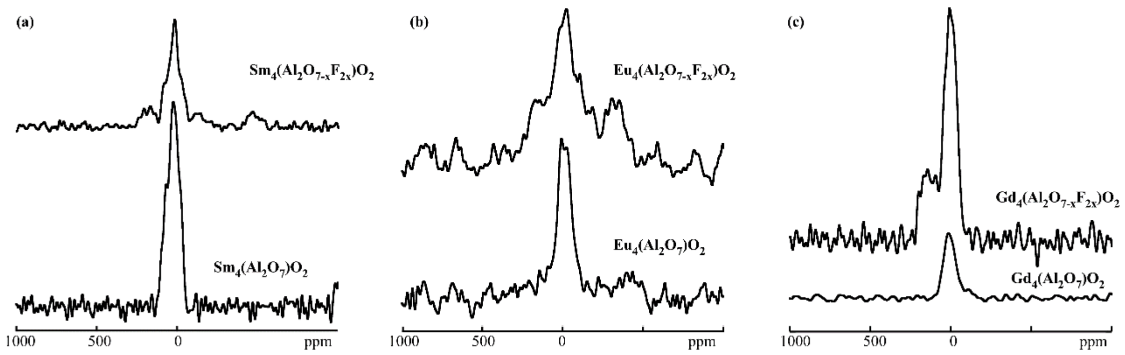

Figure S2. ^{27}Al NMR spectra (a-c) of $Ln_4(Al_2O_7)O_2$ and $Ln_4(Al_2O_6F_2)O_2$ phases in which (a) Ln=Sm, (b) Ln=Eu and (c) Ln=Gd.

Table S1. Refined cell parameters for $Sm_4(Al_2O_7□)O_2$ and $Eu_4(Al_2O_7□)O_2$ and their fluorinated derivatives in space group $P2_1/c$.

Sample	*a (Å)*	*b (Å)*	*c (Å)*	*β(º)*
$Sm_4(Al_2O_7□)O_2$	7.6356(5)	10.7312(8)	11.2307(8)	109.23(1)
$Sm_4(Al_2O_6F_2)O_2$	7.6186(8)	10.870(1)	11.107(1)	108.80(1)
$Eu_4(Al_2O_7□)O_2$	7.5891(4)	10.6833(6)	11.2014(6)	109.14(1)
$Eu_4(Al_2O_6F_2)O_2$	7.5885(7)	10.839(1)	11.077(1)	108.78(1)

Table S2. Refined positional parameters for $Sm_4(Al_2O_7\square)O_2$ and their fluorinated derivative in space group $P2_1/c$.

$\boldsymbol{Sm_4(Al_2O_7\square)O_2}$				$\boldsymbol{Sm_4(Al_2O_6F_2)O_2}$			
**Atom**	**x**	**y**	**z**	**Atom**	**x**	**y**	**z**
Sm1	0.531(2)	0.0998(8)	0.7887(7)	Sm1	0.5304(7)	0.0876(5)	0.7899(4)
Sm2	0.022(2)	0.0888(8)	0.8103(7)	Sm2	0.0270(7)	0.0759(4)	0.8243(4)
Sm3	0.335(2)	0.124(1)	0.4221(9)	Sm3	0.3326(9)	0.1310(5)	0.4140(4)
Sm4	0.834(2)	0.122(1)	0.423(1)	Sm4	0.8389(7)	0.1324(5)	0.4284(4)
Al1	0.201(8)	0.176(5)	0.122(5)	Al1	0.199(1)	0.1837(7)	0.1338(8)
Al2	0.682(7)	0.183(5)	0.121(5)	Al2	0.682(1)	0.1832(8)	0.1212(7)
O1	0.79(1)	0.248(7)	0.751(8)	O1	0.790(1)	0.2381(8)	0.7523(8)
O2	0.25(1)	0.225(7)	0.764(8)	O2	0.244(1)	0.2095(7)	0.7676(9)
O3	0.19(1)	0.017(1)	0.156(7)	O3	0.194(1)	0.0166(8)	0.156(2)
O4	0.07(1)	0.233(8)	0.976(7)	O4	0.085(3)	0.230(2)	0.979(1)
O5	0.41(1)	0.224(5)	0.107(6)	F1	0.414(1)	0.2237(9)	0.1070(8)
O6	0.64(1)	0.231(9)	0.970(7)	O6	0.639(5)	0.222(2)	0.967(1)
O7	0.682(9)	0.01(1)	0.160(6)	O7	0.682(2)	0.0124(9)	0.160(2)
O8	0.08(1)	-0.008(9)	0.379(8)	O8	0.072(1)	-0.0044(8)	0.4088(7)
O9	0.54(2)	0.02(1)	0.389(8)	O9	0.536(1)	0.0217(8)	0.4004(8)
-	-	-	-	F2	0.052(1)	0.7711(9)	0.3416(8)

Table S3. Refined positional parameters for $Eu_4(Al_2O_7\square)O_2$ and their fluorinated derivative in space group $P2_1/c$.

$\boldsymbol{Eu_4(Al_2O_7\square)O_2}$				$\boldsymbol{Eu_4(Al_2O_6F_2)O_2}$			
**Atom**	**x**	**y**	**z**	**Atom**	**x**	**y**	**z**
Eu1	0.5268(7)	0.0994(5)	0.7892(4)	Eu1	0.5336(7)	0.0850(5)	0.7979(4)
Eu2	0.0256(7)	0.0929(4)	0.8089(4)	Eu2	0.0252(7)	0.0759(4)	0.8236(4)
Eu3	0.3343(8)	0.1233(5)	0.4228(4)	Eu3	0.3323(8)	0.1313(5)	0.4146(5)
Eu4	0.8349(7)	0.1221(5)	0.4224(5)	Eu4	0.8367(7)	0.1303(4)	0.4242(4)
Al1	0.192(1)	0.1818(8)	0.1171(7)	Al1	0.194(1)	0.2046(7)	0.1270(7)
Al2	0.690(1)	0.1776(7)	0.1351(7)	Al2	0.690(1)	0.1776(7)	0.1351(7)
O1	0.790(1)	0.2482(8)	0.7508(8)	O1	0.784(3)	0.230(1)	0.7590(9)
O2	0.246(1)	0.2252(8)	0.7644(8)	O2	0.246(1)	0.2028(7)	0.7727(9)
O3	0.194(2)	0.017(1)	0.156(4)	O3	0.191(2)	0.0357(9)	0.136(3)
O4	0.071(3)	0.233(2)	0.9759(9)	O4	0.073(3)	0.228(2)	0.976(1)
O5	0.414(1)	0.224(2)	0.107(2)	F1	0.414(2)	0.224(3)	0.107(2)
O6	0.644(3)	0.231(2)	0.9704(8)	O6	0.662(3)	0.214(1)	0.9808(8)
O7	0.682(2)	0.012(1)	0.160(3)	O7	0.682(2)	0.0124(8)	0.160(2)
O8	0.075(1)	-0.0082(9)	0.3790(7)	O8	0.074(1)	-0.0051(8)	0.3783(7)
O9	0.537(1)	0.0222(8)	0.3890(8)	O9	0.532(1)	0.0201(8)	0.3961(8)
-	-	-	-	F2	0.051(1)	0.7606(9)	0.3570(8)

Table S4. Selected bond distances (diagonal) (Å) and angles (º) for $Sm_4(Al_2O_7)O_2$ from the full structural refinement.

**Sm(1)**	O(1)	O(2)	O(3)	O(5)	O(6)	O(7)	O(9)
O(9)	99.61(1)	105.88(1)	81.47(1)	79.64(1)	171.69(2)	86.64(1)	**2.298(1)**
O(7)	173.75(2)	68.82(1)	107.91(2)	118.84(2)	100.82(1)	**2.243(1)**	
O(6)	72.96(1)	80.46(1)	92.66(1)	99.65(2)	**2.394(1)**		
O(5)	62.68(1)	58.89(1)	127.88(2)	**2.710(1)**			
O(3)	73.44(1)	171.46(2)	**2.344(1)**				
O(2)	108.94(2)	**2.494(1)**					
O(1)	**2.680(1)**						

**Sm(2)**	O(1)	O(2)	O(3)	O(4)	O(7)	O(8)
O(8)	91.67(1)	93.91(1)	85.91(1)	160.75(2)	87.51(2)	**2.186(1)**
O(7)	158.04(2)	67.11(1)	118.55(2)	106.70(1)	**2.428(1)**	
O(4)	70.26(1)	80.30(1)	98.05(1)	**2.351(1)**		
O(3)	83.24(1)	174.31(2)	**2.137(1)**			
O(2)	91.08(1)	**2.430(1)**				
O(1)	**2.395(1)**					

**Sm(3)**	O(2)	O(4)	O(5)	O(6)	O(8)	O(9)	O(9)´
O(9)´	172.77(2)	105.49(2)	78.16(1)	96.65(1)	82.47(1)	73.56(1)	**2.556(1)**
O(9)	106.53(2)	177.45(2)	120.23(2)	71.42(1)	106.42(2)	**2.020(1)**	
O(8)	104.25(2)	75.69(1)	120.67(2)	177.83(2)	**2.355(1)**		
O(6)	76.72(1)	106.47(2)	60.94(1)	**2.731(1)**			
O(5)	95.89(2)	57.22(1)	**2.556(1)**				
O(4)	74.10(1)	**2.755(1)**					
O(2)	**2.332(1)**						

*O(9)´: -x, -y, -z

**Sm(4)**	O(1)	O(4)	O(6)	O(8)	O(8)´	O(9)
O(9)	105.27(1)	162.08(2)	73.27(1)	115.77(2)	83.70(2)	**2.424(1)**
O(8)´	170.41(2)	97.96(1)	98.13(1)	83.73(1)	**2.433(1)**	
O(8)	95.11(1)	82.12(1)	170.95(2)	**2.483(1)**		
O(6)	81.58(1)	88.85(1)	**2.320(1)**			
O(4)	72.46(1)	**2.310(1)**				
O(1)	**2.311(1)**					

*O(8)´: -x, -y, -z

**Al(1)**	O(2)	O(3)	O(4)	O(5)
O(5)	90.24(1)	113.40(2)	93.60(2)	**1.768(1)**
O(4)	120.11(2)	120.09(2)	**1.729(1)**	
O(3)	112.48(2)	**1.755(1)**		
O(2)	**1.851(1)**			

**Al(2)**	O(1)	O(5)	O(6)	O(7)
O(7)	105.98(2)	98.65(2)	120.76(2)	**1.884(1)**
O(6)	129.49(3)	91.24(1)	**1.701(1)**	
O(5)	100.15(2)	**2.041(1)**		
O(1)	**1.596(1)**			

Table S5. Bond-valence for $Sm_4(Al_2O_7)O_2$ phase calculated from the Rietveld refinement.

	Sm(1)	Sm(2)	Sm(3)	Sm(4)	Al(1)	Al(2)	
O(1)	0.20(1)	0.44(1)		0.55(1)		1.07(1)	2.25(1)
O(2)	0.33(1)	0.40(1)	0.52(1)		0.54(1)		1.78(1)
O(3)	0.50(1)	0.88(1)			0.69(1)		2.07(1)
O(4)		0.49(1)	0.16(1)	0.55(1)	0.75(1)		1.95(1)
O(5)	0.19(1)		0.28(1)		0.67(1)	0.32(1)	1.46(1)
O(6)	0.44(1)		0.18(1)	0.53(1)		0.80(1)	1.95(1)
O(7)	0.66(1)	0.40(1)				0.49(1)	1.55(1)
O(8)		0.77(1)	0.49(1)	0.34(1)			
O(8)´				0.39(1)			1.99(1)
O(9)	0.57(1)		1.20(1)	0.40(1)			
O(9)´			0.28(1)				2.45(1)
	2.88(1)	3.37(1)	3.11(1)	2.77(1)	2.65(1)	2.68(1)	Valence

*O(8)´: -x, -y, -z; O(9)´: -x, -y, -z

Table S6. Selected bond distances (diagonal) (Å) and angles (°) for $Sm_4(Al_2O_6F_2)O_2$ from the full structural refinement.

**Sm(1)**	O(1)	O(2)	O(3)	F(1)	O(6)	O(7)	O(9)
O(9)	97.13(1)	104.63(1)	84.16(2)	77.98(1)	168.41(2)	90.78(1)	**2.334(1)**
O(7)	171.48(2)	65.77(1)	112.49(2)	117.53(2)	100.74(1)	**2.166(1)**	
O(6)	71.30(1)	79.30(1)	92.52(1)	95.34(2)	**2.380(1)**		
F(1)	61.53(1)	58.78(1)	126.67(2)	**2.821(1)**			
O(3)	71.67(1)	170.91(2)	**2.286(1)**				
O(2)	108.88(2)	**2.496(1)**					
O(1)	**2.701(1)**						

**Sm(2)**	O(1)	O(2)	O(3)	O(4)	O(7)	O(8)	F(2)
F(2)	44.14(1)	54.02(1)	114.32(1)	90.63(2)	109.36(2)	61.25(1)	**2.410(1)**
O(8)	86.80(1)	85.51(1)	88.60(1)	151.87(2)	84.98(2)	**2.573(1)**	
O(7)	152.01(2)	64.06(1)	125.46(2)	106.61(1)	**2.369(1)**		
O(4)	70.89(1)	77.30(1)	103.98(1)	**2.339(1)**			
O(3)	80.94(1)	168.33(2)	**2.035(1)**				
O(2)	88.67(1)	**2.431(1)**					
O(1)	**2.465(1)**						

**Sm(3)**	O(2)	O(4)	F(1)	O(6)	O(8)	O(9)	O(9)´
O(9)´	171.17(2)	105.68(1)	78.57(1)	96.75(1)	74.73(1)	64.41(1)	**2.584(1)**
O(9)	116.64(2)	169.26(2)	116.28(1)	74.52(1)	106.52(2)	**1.997(1)**	
O(8)	112.32(1)	73.21(1)	111.31(2)	169.38(2)	**2.460(2)**		
O(6)	75.61(1)	103.73(2)	59.99(1)	**2.728(1)**			
F(1)	93.59(2)	55.46(1)	**2.573(1)**				
O(4)	72.47(1)	**2.693(1)**					
O(2)	**2.323(1)**						

*O(9)´: -x, -y, -z

**Sm(4)**	O(1)	O(4)	O(6)	O(8)	O(8)´	O(9)	F(2)
F(2)	119.10(2)	61.94(1)	64.63(1)	109.01(1)	62.58(1)	107.38(2)	**2.636(1)**
O(9)	106.86(1)	165.68(2)	73.71(1)	111.61(2)	80.21(1)	**2.531(1)**	
O(8)´	170.88(2)	101.22(1)	108.84(1)	68.75(1)	**2.208(1)**		
O(8)	102.85(1)	81.85(1)	173.14(2)	**2.3758(2)**			
O(6)	79.08(1)	92.50(1)	**2.329(1)**				
O(4)	73.41(1)	**2.323(1)**					
O(1)	**2.341(1)**						

*O(8)´: -x, -y, -z

**Al(1)**	O(2)	O(3)	O(4)	F(1)	F(2)
F(2)	64.77(1)	110.64(2)	79.81(1)	140.84(2)	**2.227(1)**
F(1)	92.17(1)	108.41(1)	87.60(2)	**1.814(1)**	
O(4)	121.70(2)	113.36(2)	**1.728(1)**		
O(3)	121.68(2)	**1.835(1)**			
O(2)	**1.829(1)**				

**Al(2)**	O(1)	F(1)	O(6)	O(7)	F(2)
F(2)	55.51(1)	140.64(2)	86.86(2)	117.04(2)	**2.159(1)**
O(7)	109.78(2)	99.00(1)	117.50(2)	**1.907(1)**	
O(6)	129.56(2)	90.24(1)	**1.688(1)**		
F(1)	99.12(2)	**2.040(1)**			
O(1)	**1.660(1)**				

Table S7. Bond-valence for $Sm_4(Al_2O_6F_2)O_2$ phase calculated from Rietveld refinement.

	Sm(1)	Sm(2)	Sm(3)	Sm(4)	Al(1)	Al(2)	
O(1)	0.19(1)	0.36(1)		0.50(1)		0.90(1)	1.96(1)
O(2)	0.33(1)	0.40(1)	0.53(1)		0.57(1)		1.82(1)
O(3)	0.59(1)	1.15(1)			0.56(1)		2.30(1)
O(4)		0.51(1)	0.20(1)	0.53(1)	0.75(1)		1.98(1)
F(1)	0.11(1)		0.21(1)		0.48(1)	0.26(1)	1.06(1)
O(6)	0.45(1)		0.18(1)	0.52(1)		0.83(1)	1.98(1)
O(7)	0.81(1)	0.47(1)				0.46(1)	1.74(1)
O(8)		0.27(1)	0.37(1)	0.46(1)			
O(8)´				0.72(1)			1.82(1)
O(9)	0.51(1)		1.28(1)	0.30(1)			
O(9)´			0.26(1)				2.36(1)
F(2)		0.31(1)		0.18(1)	0.16(1)	0.19(1)	0.83(1)
	3.00(1)	3.46(1)	3.01(1)	3.22(1)	2.52(1)	2.64(1)	Valence

*O(8)´: -x, -y, -z; O(9)´: -x, -y, -z

Table S8. Selected bond distances (diagonal) (Å) and angles (°) for $Eu_4(Al_2O_7)O_2$ from the full structural refinement.

**Eu(1)**	O(1)	O(2)	O(3)	O(5)	O(6)	O(7)	O(9)
O(9)	98.72(1)	106.45(1)	81.02(1)	79.35(1)	170.20(1)	87.23(1)	**2.296(1)**
O(7)	174.02(1)	69.77(1)	107.99(1)	119.80(1)	101.55(1)	**2.203(1)**	
O(6)	72.48(1)	80.94(1)	92.03(1)	99.65(1)	**2.388(1)**		
O(5)	62.26(1)	59.06(1)	126.77(1)	**2.706(1)**			
O(3)	72.71(1)	171.86(1)	**2.353(1)**				
O(2)	108.68(1)	**2.458(1)**					
O(1)	**2.693(1)**						

**Eu(2)**	O(1)	O(2)	O(3)	O(4)	O(7)	O(8)
O(8)	91.83(1)	95.28(1)	84.56(1)	162.12(1)	87.64(1)	**2.186(1)**
O(7)	160.65(1)	68.18(1)	116.61(1)	107.28(1)	**2.407(1)**	
O(4)	70.80(1)	81.71(1)	96.96(1)	**2.331(1)**		
O(3)	82.55(1)	175.18(1)	**2.178(1)**			
O(2)	92.64(1)	**2.363(1)**				
O(1)	**2.370(1)**					

**Eu(3)**	O(2)	O(4)	O(5)	O(6)	O(8)	O(9)	O(9)´
O(9)´	172.65(1)	105.80(1)	78.21(1)	96.60(1)	82.80(1)	73.68(1)	**2.542(1)**
O(9)	106.04(1)	177.41(1)	120.16(1)	71.30(1)	106.35(1)	**2.015(1)**	
O(8)	104.17(1)	76.02(1)	121.15(1)	177.64(1)	**2.336(1)**		
O(6)	76.53(1)	106.34(1)	60.83(1)	**2.723(1)**			
O(5)	95.95(1)	57.32(1)	**2.545(1)**				
O(4)	74.14(1)	**2.734(1)**					
O(2)	**2.332(1)**						

*O(9)´: -x, -y, -z

**Eu(4)**	O(1)	O(4)	O(6)	O(8)	O(8)´	O(9)
O(9)	105.12(1)	161.81(1)	73.20(1)	115.73(1)	83.63(1)	**2.415(1)**
O(8)´	170.52(1)	97.90(1)	97.88(1)	83.84(1)	**2.429(1)**	
O(8)	95.33(1)	82.42(1)	171.07(1)	**2.464(1)**		
O(6)	81.52(1)	88.66(1)	**2.314(1)**			
O(4)	72.65(1)	**2.296(1)**				
O(1)	**2.302(1)**					

*O(8)´: -x, -y, -z

**Al(1)**	O(2)	O(3)	O(4)	O(5)
O(5)	89.18(1)	108.95(1)	95.56(1)	**1.784(1)**
O(4)	124.73(1)	120.91(1)	**1.640(1)**	
O(3)	108.96(1)	**1.816(1)**		
O(2)	**1.854(1)**			

**Al(2)**	O(1)	O(5)	O(6)	O(7)
O(7)	114.79(1)	99.92(1)	117.13(1)	**1.791(1)**
O(6)	125.27(1)	85.60(1)	**1.851(1)**	
O(5)	101.63(1)	**2.074(1)**		
O(1)	**1.496(1)**			

Table S9. Bond-valence for $Eu_4(Al_2O_7)O_2$ phase calculated from Rietveld refinement.

	Eu(1)	Eu(2)	Eu(3)	Eu(4)	Al(1)	Al(2)	
O(1)	0.19(1)	0.45(1)		0.54(1)		1.40(1)	2.58(1)
O(2)	0.35(1)	0.46(1)	0.50(1)		0.53(1)		1.84(1)
O(3)	0.47(1)	0.76(1)			0.59(1)		1.81(1)
O(4)		0.50(1)	0.17(1)	0.55(1)	0.95(1)		2.16(1)
O(5)	0.18(1)		0.28(1)		0.64(1)	0.29(1)	1.40(1)
O(6)	0.43(1)		0.17(1)	0.52(1)		0.54(1)	1.66(1)
O(7)	0.70(1)	0.41(1)				0.63(1)	1.74(1)
O(8)		0.74(1)	0.49(1)	0.35(1)			
O(8)´				0.38(1)			1.96(1)
O(9)	0.55(1)		1.17(1)	0.40(1)			
O(9)´			0.28(1)				2.40(1)
	2.88(1)	3.31(1)	3.07(1)	2.74(1)	2.71(1)	2.86(1)	Valence

*O(8)´: -x, -y, -z; O(9)´: -x, -y, -z

Table S10. Selected bond distances (diagonal) (Å) and angles (°) for $Eu_4(Al_2O_6F_2)O_2$ from the full structural refinement.

**Eu(1)**	O(1)	O(2)	O(3)	F(1)	O(6)	O(7)	O(9)
O(9)	95.26(1)	103.27(1)	85.37(1)	75.73(1)	165.69(2)	90.45(1)	**2.340(1)**
O(7)	172.52(2)	64.17(1)	108.96(2)	115.78(1)	103.82(1)	**2.121(1)**	
O(6)	70.61(1)	84.24(1)	88.96(1)	98.46(2)	**2.394(1)**		
F(1)	61.32(1)	59.28(1)	131.06(2)	**2.891(1)**			
O(3)	76.39(1)	168.63(2)	**2.376(1)**				
O(2)	109.66(2)	**2.468(1)**					
O(1)	**2.609(1)**						

**Eu(2)**	O(1)	O(2)	O(3)	O(4)	O(7)	O(8)	F(2)
F(2)	48.58(1)	54.42(1)	122.57(2)	91.14(2)	107.67(1)	63.05(1)	**2.595(1)**
O(8)	87.84(1)	89.84(1)	88.91(1)	153.52(2)	86.71(1)	**2.252(1)**	
O(7)	154.81(2)	62.41(1)	120.28(2)	108.09(1)	**2.370(1)**		
O(4)	69.34(1)	78.56(1)	101.44(1)	**2.309(1)**			
O(3)	84.16(1)	176.95(2)	**2.194(1)**				
O(2)	93.02(1)	**2.369(1)**					
O(1)	**2.408(1)**						

**Eu(3)**	O(2)	O(4)	F(1)	O(6)	O(8)	O(9)	O(9)´
O(9)´	168.50(2)	106.22(1)	77.84(1)	94.36(1)	80.52(1)	65.48(1)	**2.600(1)**
O(9)	116.97(2)	171.55(2)	117.84(1)	75.78(1)	102.58(1)	**1.997(1)**	
O(8)	108.98(1)	76.69(1)	119.22(2)	174.82(2)	**2.384(1)**		
O(6)	76.02(1)	104.18(1)	58.40(1)	**2.907(1)**			
F(1)	91.60(2)	56.83(1)	**2.558(1)**				
O(4)	70.83(1)	**2.740(1)**					
O(2)	**2.340(1)**						

*O(9)´: -x, -y, -z

**Eu(4)**	O(1)	O(4)	O(6)	O(8)	O(8)´	O(9)	F(2)
F(2)	111.34(2)	57.37(1)	57.69(1)	114.67(1)	60.54(1)	110.15(2)	**2.582(1)**
O(9)	107.18(1)	163.41(2)	78.58(1)	112.80(2)	80.16(1)	**2.532(1)**	
O(8)´	171.06(2)	100.06(1)	100.13(1)	81.23(1)	**2.477(1)**		
O(8)	100.09(1)	83.47(1)	168.55(2)	**2.499(1)**			
O(6)	76.83(1)	85.09(1)	**2.353(1)**				
O(4)	71.42(1)	**2.289(1)**					
O(1)	**2.309(1)**						

*O(8)´: -x, -y, -z

**Al(1)**	O(2)	O(3)	O(4)	F(1)	F(2)
**F(2)**	72.54(1)	105.53(1)	79.40(1)	155.66(2)	**2.012(1)**
**F(1)**	95.88(1)	98.80(1)	95.34(2)	**1.771(1)**	
**O(4)**	134.42(2)	101.52(2)	**1.644(1)**		
**O(3)**	120.05(2)	**1.834(1)**			
**O(2)**	**1.833(1)**				
**Al(2)**	O(1)	F(1)	O(6)	O(7)	F(2)
F(2)	64.44(1)	140.72(2)	76.12(1)	119.07(2)	**2.137(1)**
O(7)	119.59(2)	100.21(1)	112.52(2)	**1.816(1)**	
O(6)	124.96(2)	90.00(1)	**1.698(1)**		
F(1)	96.99(2)	**2.078(1)**			
O(1)	**1.664(1)**				

Table S11. Bond-valence for $Eu_4(Al_2O_6F_2)O_2$ phase calculated from Rietveld refinement.

	Eu(1)	Eu(2)	Eu(3)	Eu(4)	Al(1)	Al(2)	
O(1)	0.24(1)	0.41(1)		0.53(1)		0.89(1)	2.06(1)
O(2)	0.34(1)	0.45(1)	0.49(1)		0.56(1)		1.84(1)
O(3)	0.44(1)	0.72(1)			0.56(1)		1.79(1)
O(4)		0.53(1)	0.16(1)	0.56(1)	0.94(1)		2.19(1)
F(1)	0.08(1)		0.20(1)		0.54(1)	0.24(1)	1.06(1)
O(6)	0.42(1)		0.10(1)	0.47(1)		0.81(1)	1.81(1)
O(7)	0.88(1)	0.45(1)				0.59(1)	1.99(1)
O(8)		0.62(1)	0.43(1)	0.32(1)			
O(8)´				0.34(1)			1.70(1)
O(9)	0.49(1)		1.23(1)	0.29(1)			
O(9)´			0.24(1)				2.25(1)
F(2)		0.18(1)		0.19(1)	0.28(1)	0.20(1)	0.85(1)
	2.89(1)	3.36(1)	2.86(1)	2.69(1)	2.89(1)	2.72(1)	Valence

*O(8)´: -x, -y, -z; O(9)´: -x, -y, -z
